# Nanoscale control of coherency stress in Ni–Pd interfaces through grading and ternary buffer layers

**DOI:** 10.1039/d6na00262e

**Published:** 2026-06-26

**Authors:** Emmanuel-Peters Teke Tebo, Sina Karimzadeh, Tien-Chien Jen

**Affiliations:** a Department of Mechanical Engineering Science, University of Johannesburg Gauteng 2006 South Africa 221191307@student.uj.ac.za skarimzadeh@uj.ac.za tjen@uj.ac.za

## Abstract

Pd-based hydrogen separation membranes often develop residual coherency stresses at interfaces because of lattice mismatch with adjacent metallic layers, which can compromise structural stability. This study uses molecular dynamics simulations to investigate how interface architecture controls stress accommodation and defect-mediated relaxation in three representative systems: a sharp Ni|Pd interface, a compositionally graded Ni–Pd interface, and a ternary Ni–Co–Pd interface incorporating a Co buffer layer. All architectures were evaluated under identical crystallographic, thermodynamic, and interatomic-potential conditions at 300 K to isolate geometric effects. The sharp Ni|Pd architecture confines the ∼10.5% lattice mismatch within an atomically narrow region and produces highly localized residual stresses, reaching −9.1 GPa compressive and 2.8 GPa tensile. Compositional grading redistributes the mismatch over a broader transition region and lowers the peak tensile stress by 43%, while the Co-buffered ternary architecture further reduces the peak compressive stress by 53% through strain partitioning. Defect screening using centrosymmetry parameter (CSP), polyhedral template matching (PTM), and dislocation extraction analysis (DXA) shows that relaxation occurs through defect-mediated accommodation rather than ideal coherent behavior. The ternary architecture exhibits the strongest suppression of defect-like signatures, reducing the mean HCP-classified fraction from 14.3% to 5.0% and decreasing DXA-detectable line length from 427.8 to 199.1 nm. These results demonstrate that nanoscale grading and intermediate buffer layers effectively suppress residual coherency-stress localization and provide a mechanical route for improving structural stability in lattice-mismatched metallic interfaces.

## Abstract 2

Interface architecture controls Ni–Pd coherency-stress distribution. Grading and Co buffering reduce peak stresses and local disorder in Pd-based hydrogen membrane interfaces.

## Graphical abstract

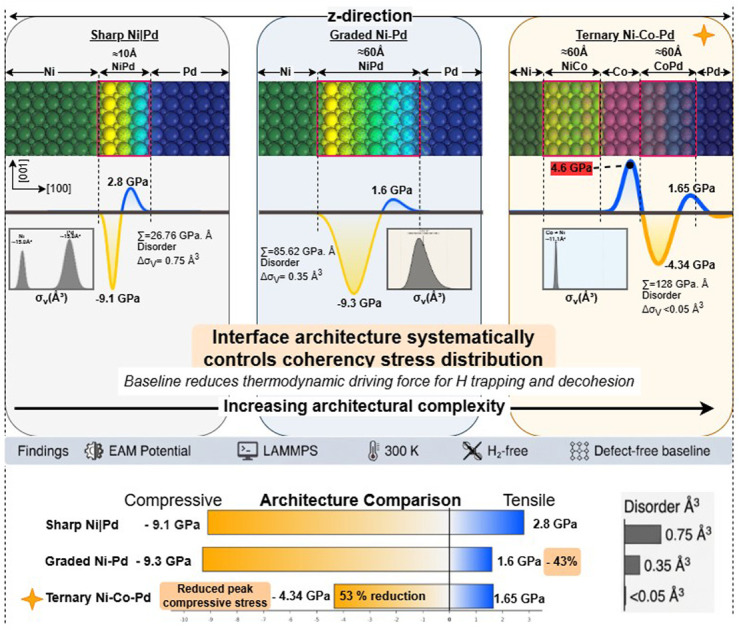

## 1 Introduction

Palladium-based metallic membranes remain central to high-purity hydrogen separation, particularly in integrated steam methane reforming (SMR) systems.^1–4^ Although these membranes exhibit excellent selectivity, their long-term mechanical durability is compromised by high-temperature operation. Prolonged thermal exposure promotes interdiffusion between the Pd-selective layer and adjacent supports or interlayers,^5–10^ driving intermetallic formation, interface roughening, and morphological evolution.^11–14^ These changes correlate with reduced mechanical integrity and shortened service lifetimes.^7,15,16^

A critical yet under-quantified feature of these membrane stacks is the support/selective-layer interface. When the compositional transition is abrupt, the lattice parameter mismatch between adjoining layers introduces substantial elastic incompatibility.^17–20^ Classical semi-coherent interface mechanics predicts that such incompatibility must be accommodated within a narrow region, generating intense coherency stresses that often relax through misfit dislocation formation, stacking-fault networks, and interface roughening.^19,21–24^ These localized stress concentrations define where damage initiates and how the interface evolves under thermal and chemical cycling.^25–29^

Despite this established framework, the intrinsic mechanical role of coherency stresses in Ni–Pd membrane stacks remains insufficiently isolated. Most membrane studies evaluate interfacial mechanics in the presence of hydrogen, thermal cycling, or reactive environments,^5,30^ making it difficult to extract an architecture-dependent mechanical baseline. This gap is particularly significant because Ni–Pd interfaces exhibit a large ∼10.5% lattice mismatch, arising fundamentally from the difference between the lattice constants of Ni (*a* ≈ 3.52 Å) and Pd (*a* ≈ 3.89 Å) far exceeding the modest mismatch typical of structural multilayers such as Cu–Ni (∼2.6%).^22,26,27,31–33^ Stress magnitudes, localization widths, and defect accommodation mechanisms reported in low-mismatch systems therefore cannot be extrapolated to membrane-relevant architectures without explicit re-evaluation.

Large FCC lattice mismatch cannot always be treated as a purely elastic coherency problem.^22,34^ When the film or transition-region thickness exceeds the critical thickness for coherent accommodation, mismatch relaxation can occur through localized defect-mediated mechanisms such as planar stacking-fault-like accommodation.^19,35,36^ Therefore, highly mismatched Ni–Pd interfaces should be evaluated using coupled mechanical and structural descriptors rather than stress profiles alone. In this work, the interface architectures are assessed using both residual coherency-stress fields and post-processed defect-screening metrics, including CSP, PTM, and DXA.^37–39^ This combined approach allows the sharp, graded, and ternary architectures to be compared in terms of stress localization, local lattice disorder, and HCP-like planar defect signatures.

Parallel advances in multilayer mechanics provide important insight. Atomistic studies consistently show that interface character governs dislocation nucleation, slip transmission, and plasticity.^37,40–43^ In coherent or semi-coherent interfaces, residual coherency stresses bias the onset of plasticity, while misfit dislocation networks act as preferential nucleation sites or barriers depending on their geometry.^18,44–46^ In graded metallic systems, compositional gradients systematically tune stress fields and defect behavior by distributing elastic incompatibility across multiple atomic planes.^47–51^ These studies collectively demonstrate that interface morphology not chemistry controls the spatial distribution of strain energy.

Functionally graded interfaces (FGIs) therefore offer a promising route to mitigate stress localization in membrane stacks.^49,50,52^ Advances in thin-film deposition, particularly atomic layer deposition (ALD), now enable angstrom-scale control of composition,^53–57^ making graded architectures experimentally accessible. Yet, the membrane literature remains predominantly performance-driven, emphasizing permeation flux, selectivity, and impurity tolerance,^58,59^ while treating the interface as a binary intact/failed entity rather than a spatially distributed mechanical system. The intrinsic stress fields that precede hydrogen-coupled degradation remain largely unquantified.^17,45,55,60^

This work addresses this gap by isolating interface architecture as an independent mechanical variable in the as-fabricated state. Using atomistic simulations, sharp Ni|Pd, graded Ni–Pd, and ternary Ni–Co–Pd architectures are compared under identical crystallography, interatomic potential, thermostatting protocol, and boundary conditions at 300 K. The relaxed structures are not interpreted as ideal defect-free coherent interfaces. Instead, they are treated as coherency-dominated interface states in which residual elastic stress may coexist with localized defect-mediated relaxation. Differences among the sharp, graded, and ternary architectures are therefore evaluated using both stress profiles and structural diagnostics, including Voronoi volume fluctuations, CSP, PTM, and DXA. By excluding hydrogen and thermal mismatch in the present study, the simulations establish a mechanical baseline for the residual stress and defect-relaxation landscape upon which future hydrogen-coupled and thermomechanical degradation mechanisms may act.

In summary, while lattice mismatch is dictated by crystallography, the spatial distribution of strain energy is demonstrated to be an architectural choice. This study evaluates how interface morphology governs coherency stress redistribution in Ni–Pd based membrane stacks and provides a fundamental lattice-level boundary condition for future hydrogen-coupled simulations and *operando* durability assessments.

## 2 Methodology

### 2.1 Construction of interface architectures

To isolate the intrinsic effect of architectural geometry on stress redistribution, all membrane architectures were constructed on a face-centered cubic (FCC) Ni substrate oriented along the [001] direction.^25,33,56,61^ This low-index orientation provides a well-defined crystallographic reference frame and minimizes orientation-dependent artifacts, such as partial dislocation emission from faceted boundaries. A periodic slab geometry was employed in the in-plane directions (*x*, *y*) to eliminate grain boundaries and edge effects, ensuring that the compositional gradient remained the sole structural variable. Each simulation cell contained approximately 105 000 atoms, with initial in-plane dimensions of 7.04 × 7.04 nm^2^ and a total thickness of 24.11 nm. For the binary architectures (sharp and graded Ni–Pd), the constituent metals were distributed in an approximately equiatomic global ratio of 50% Ni and 50% Pd. For the ternary Ni–Co–Pd architecture, the volumetric fractions were deliberately partitioned (approximately 26% Ni, 40% Co, and 34% Pd) to accommodate the geometric requirements of the dual transitional gradients. These requirements include a Ni-to-Co compositional ramp, a central pure-Co buffer region, and a Co-to-Pd compositional ramp of comparable spatial extent. This partition was selected as a representative configuration to demonstrate the structural quiet-zone concept and to evaluate how an intermediate buffer layer redistributes coherency stress.^48,49^ As demonstrated in several computational studies, systematic optimization of buffer thickness, ramp width, and composition to maximize peak-stress suppression requires dedicated algorithmic modeling^48,49,62–64^ and is therefore identified as a target for future work.

**Fig. 1:**
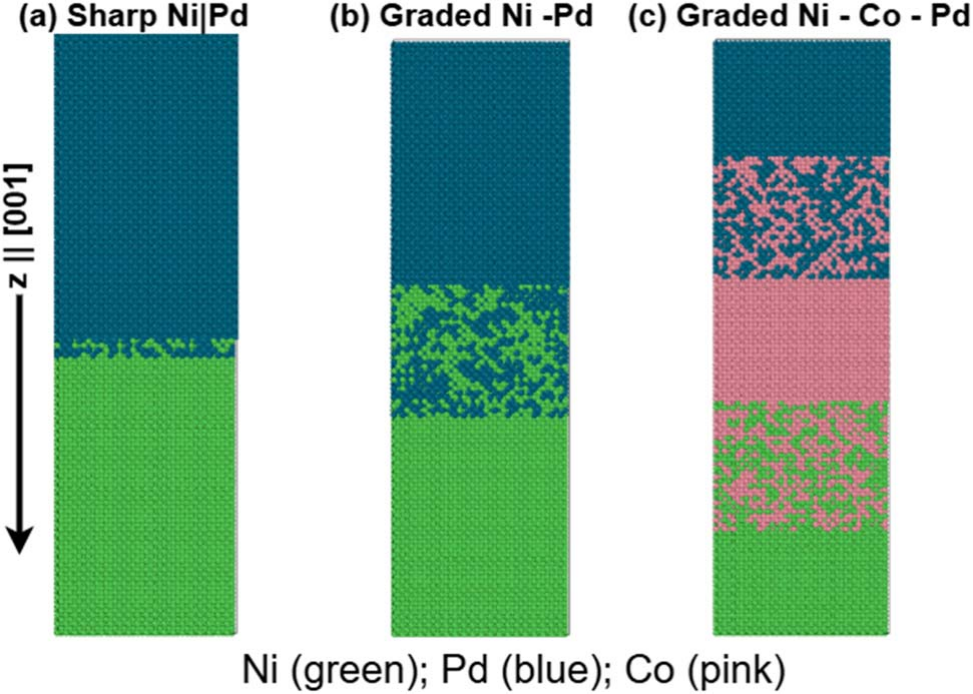
Atomistic model geometries at initial construction on an FCC Ni(001) substrate: (a) sharp Ni|Pd interface, (b) graded Ni–Pd interface, and (c) graded Ni–Co–Pd interface used to test architectural robustness under alloy extension. Colors indicate species: Ni (green), Pd (blue), and Co (pink). The interface normal is along *z*‖[001].

Three interface architectures (Fig. 1) were examined: a sharp Ni|Pd interface, a binary graded Ni–Pd interface, and a ternary graded Ni–Co–Pd interface. In the graded systems, composition was varied through discrete, layer-wise increments across successive atomic planes. This “digital grading” approach mirrors the layer-by-layer compositional control achievable *via* ALD,^54,65^ and preserves FCC lattice continuity without introducing amorphization. The sharp interface confined the total ∼10.5 lattice mismatch to a transition region of ∼7 Å, whereas the graded architectures distributed the same mismatch over ∼53 Å along the interface normal (*z*).

### 2.2 Simulation framework

Classical molecular dynamics (MD) simulations were performed using LAMMPS.^66^ Interatomic interactions were described using the Embedded Atom Method (EAM) alloy potential of Zhou *et al.* (2004). This potential was selected for its proven accuracy in reproducing the lattice constants (*a*_Ni_ = 3.52 Å, *a*_Pd_ = 3.89 Å), elastic moduli, and stacking fault energetics required to capture the coherency strain fields of these specific transition metals.^23,51,55^ Periodic boundary conditions were applied along *x* and *y*, while a non-periodic, shrink-wrapped boundary was used along *z*.^68^ Crucially, no barostat or constraint was applied along *z*, enforcing a traction-free condition (*P*_*zz*_ ≈ 0). This allows for unconstrained lattice expansion perpendicular to the interface, mimicking the relaxation of a thin film with a free surface.^55,69,70^

Pressure control was applied only in-plane (*P*_*xx*_ ≈ *P*_*yy*_ ≈ 0) using an NPT ensemble at 300 K. This temperature and ensemble choice is deliberate: simulating at 300 K isolates the as-fabricated residual stress state prior to the introduction of high-temperature thermal cycling, while full 3D barostatting would artificially redistribute coherency stresses. Conversely, the semi-anisotropic NPT ensemble preserves the intrinsic mismatch-driven stress state characteristic of epitaxial growth.^40,45,51,61,67^ Each system underwent energy minimization, followed by 300 ps of thermal equilibration and a 400 ps production run with a 1.0 fs timestep.

### 2.3 Stress quantification

To spatially resolve the coherency strain field, depth-resolved virial stresses were computed using the Irving–Kirkwood formulation.^71,72^ For an atomic volume *V*_*i*_, the local stress tensor *σ*_αβ_ is defined as:1
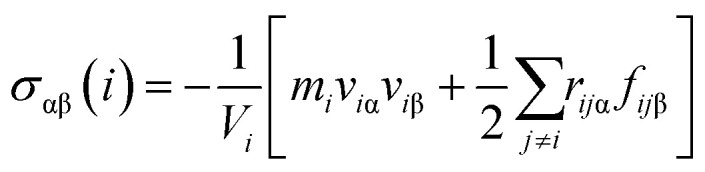
where *m*_*i*_ and *v*_*i*_ are the mass and velocity of atom *i*, and *r*_*ij*_ and *f*_*ij*_ are the relative position and force vectors between atoms *i* and *j*. To account for the significant lattice expansion across the interface, the local atomic volume *V*_*i*_ was explicitly calculated using Voronoi tessellation.^71,73,74^ The in-plane stress component *σ*_*xx*_ was selected as the primary diagnostic metric because it directly captures coherency-driven strain accommodation parallel to the interface plane, the component most relevant to dislocation nucleation in FCC multilayers.^18,26,45,75^

All tensor components (*σ*_*xx*_, *σ*_*yy*_, *σ*_*zz*_) were recorded independently without hydrostatic averaging. The spatial localization width (*w*) was quantified using the second moment of the stress profile, and the integrated stress magnitude 
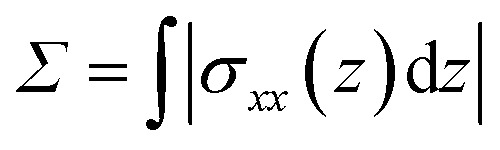
 was computed to assess the total volumetric strain energy.

### 2.4 Structural disorder metrics

Local lattice distortion was quantified using Voronoi tessellation (compute voronoi per atom). To distinguish between uniform volumetric expansion and local lattice disorder, the local-mean-centered standard deviation of Voronoi volumes, Δ*σ*_*V*_(*z*),^71,72,74,76^ was introduced as a depth-resolved scalar disorder metric:2
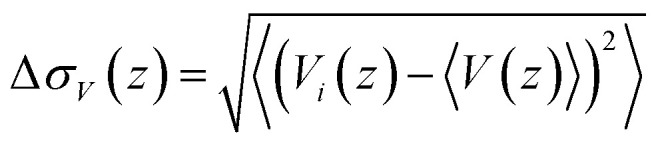
where *V*_i_(*z*) is the Voronoi volume of atom *i*in the depth bin centered at *z*, and 〈*V*(*z*)〉 denotes the local mean Voronoi volume in that bin. Angle brackets indicate averaging over atoms within the bin. Therefore, Δ*σ*_*V*_(*z*) has units of Å^3^ and quantifies local fluctuations in atomic volume after removal of the local mean volume. This metric captures local Voronoi-volume fluctuations associated with gradual compositional change and strain accommodation, providing a structural signature distinct from standard defect analysis.^44^

### 2.5 Defect-mediated relaxation screening

To determine whether local defect-mediated relaxation accompanied the residual coherency stress fields, representative production-tail snapshots were post-processed using the Centrosymmetry Parameter (CSP),^77^ Polyhedral Template Matching (PTM),^78^ and Dislocation Extraction Algorithm (DXA)^79^ implemented in OVITO.^80^ CSP was computed using 12 nearest neighbours. PTM was used to classify local atomic environments as FCC, HCP, BCC, ICO, or Other. DXA was performed using an FCC reference lattice.

PTM was used as the primary structural metric for stacking-fault-like planar relaxation because HCP-classified atomic sheets in an FCC matrix indicate local interruptions of the close-packed stacking sequence, reliably marking the presence of intrinsic stacking faults and twin boundaries.^22,26,81^ DXA was used as a corroborating line-defect diagnostic because the detected line content included multiple Burgers-vector categories, while PTM provided the more direct measure of HCP-like planar stacking interruptions.

To distinguish interfacial defect signatures from free-surface classification effects associated with the shrink-wrapped *z*-boundary, the defect-screening analysis was also performed in depth-resolved form. Atoms were binned along the interface-normal direction and separated into free-surface, interface or transition, and bulk-like interior regions. Global CSP, PTM, and DXA statistics are reported in Table S4, while depth-resolved regional statistics are reported in Table S5. Representative PTM maps are shown in Fig. S1, CSP distributions are shown in Fig. S2, and DXA Burgers-vector breakdowns are shown in Fig. S3.

### 2.6 Diagnostics, verification, and robustness checks

To ensure that the observed stress localization reflects intrinsic interface mechanics rather than numerical artifacts, a rigorous three-step validation protocol was employed. First, the consistency between local and global stress measures was evaluated by comparing spatially integrated virial stresses with the global thermodynamic pressure tensor components (*P*_*xx*_, *P*_*yy*_, and *P*_*zz*_). Regression analysis of the mechanically relevant normal stress component, *P*_*zz*_, demonstrated near-identity agreement across all three interface architectures (Table S1), with slopes ranging from approximately 0.976 to 0.998 and coefficients of determination (*R*^2^) of at least 0.976. For the barostat-controlled transverse components (*P*_*xx*_ and *P*_*yy*_), block-averaged reconstruction errors remained within the expected thermal fluctuations, indicating good agreement between local virial stress reconstruction and the global thermodynamic response.

Second, temporal convergence of the stress profiles was assessed by averaging over sliding time windows ranging from 50 to 200 ps. Convergence was considered achieved when the root-mean-square deviation between successive averaging windows decreased below 1%, indicating stable steady-state behaviour. As summarized in Table S2, the graded interface architectures reached convergence with peak-stress deviations below 2.2% at 100 ps, whereas the sharp Ni|Pd interface showed comparatively slower relaxation and retained a peak-stress deviation of approximately 3.9% over the same interval. Based on these observations, a conservative averaging period of 200 ps was selected for all reported analyses.

Finally, spatial robustness was examined by recalculating stress distributions using bin widths between 0.5 and 2.0 Å. The relative ordering of peak stress, localization width, and integrated stress remained unchanged across all tested spatial resolutions, demonstrating that the reported trends are not dependent on the selected depth discretization. As shown in Table S3, virial-conserving rebinning resulted in centroid shifts of less than 0.8 Å, corresponding to a maximum normalized displacement of 0.4 bins. These results confirm that the observed stress-localization characteristics remain spatially stable under variations in binning resolution.

## 3 Results

### 3.1 System equilibration and thermodynamic stability

**Fig. 2:**
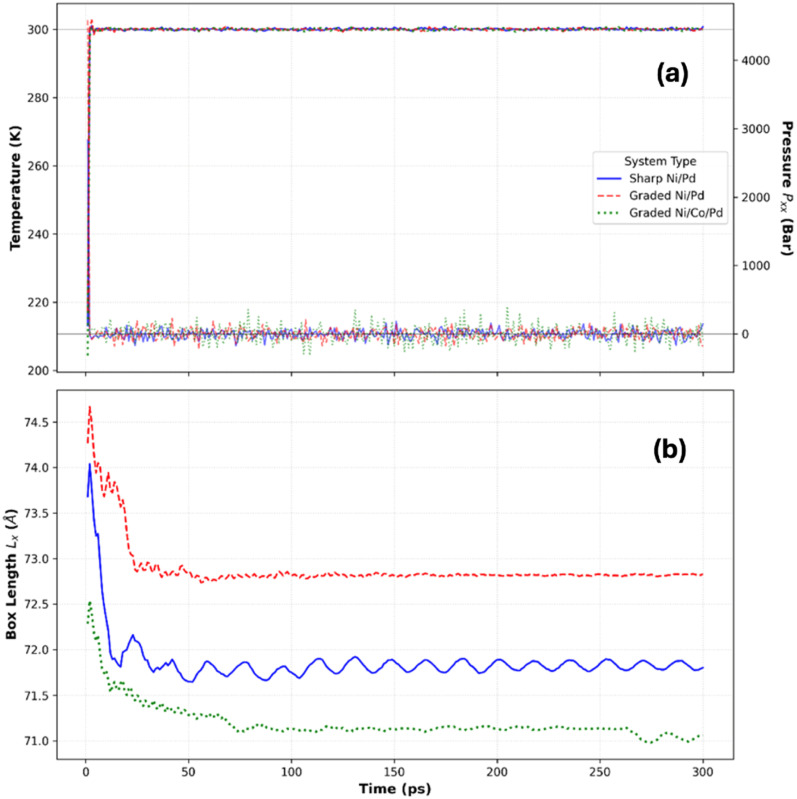
Time evolution of thermodynamic properties during 300 ps NPT equilibration. (a) Temperature and in-plane pressure (*P*_*xx*_) traces show stability at 300 K and 0 bar. (b) Simulation box length (*L*_*x*_) converges to constant values within the first 100 ps for all architectures.

To ensure that subsequent stress profiles represent steady-state intrinsic behaviour rather than transient relaxation artifacts, the thermodynamic and mechanical stability of all systems was verified. Fig. 2 displays the time evolution of temperature, in-plane pressure (*P*_*xx*_), and simulation box length (*L*_*x*_) for the sharp Ni|Pd, graded Ni–Pd, and graded Ni–Co–Pd systems during the 300 ps equilibration period.

The temperature for all three architectures (top panel) remains constant at the target value of 300 K throughout the simulation. The in-plane pressure fluctuates symmetrically around 0 bar, consistent with the target isobaric condition. The simulation box length *L*_*x*_ (bottom panel) exhibits a rapid initial adjustment during the first 50 ps, followed by stabilization. Specifically, the sharp Ni|Pd system stabilizes at approximately 71.8 Å, the graded Ni–Pd system at 72.8 Å, and the graded Ni–Co–Pd system at 71.1 Å. Beyond 100 ps, no systematic drift is observed in any thermodynamic parameter, confirming the convergence of the NPT ensemble prior to the production phase.

### 3.2 Stress localization and redistribution at sharp *versus* graded interfaces

**Fig. 3:**
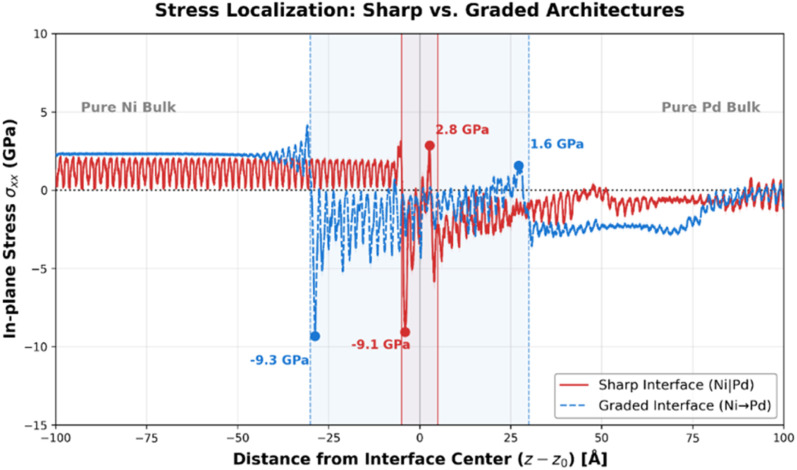
Depth-resolved in-plane stress *σ*_*xx*_(*z*) for sharp Ni|Pd (red) and graded Ni–Pd (blue) interfaces. The sharp interface shows stress peaks confined within a ±5 Å region with a maximum compressive stress of −9.1 GPa. The graded interface distributes stress over a ≈60 Å width, reaching a peak compressive stress of −9.3 GPa and a reduced tensile peak of 1.6 GPa. Shaded regions indicate the localization width (*w*).

The impact of architectural design on the internal stress field is immediately visible in the depth-resolved stress profiles. Fig. 3 presents the depth-resolved in-plane stress profiles, *σ*_*xx*_(*z*), for the sharp Ni|Pd and graded Ni–Pd interfaces at 300 K. The sharp Ni|Pd interface (red solid line) exhibits stress variation confined to a narrow region of approximately ±5 Å around the interface. A maximum compressive stress of *σ*_*xx*_(*z*) ≈ −9.1 GPa is observed, followed immediately by a tensile peak of 2.8 GPa.

To quantify the spatial extent of stress localization, the localization width (*w*) was defined using the second moment of the absolute stress profile:^24,25,49,82^3
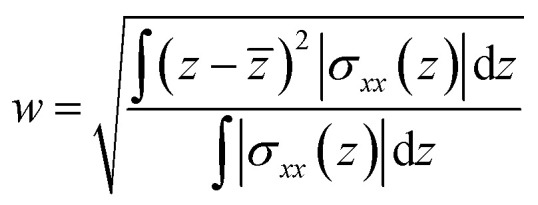
where *z̄* is the centroid of |*σ*_*xx*_(*z*)|. For the sharp interface, the calculated width is *w* = 4.4 Å.

In contrast, the graded Ni–Pd architecture (blue dashed line) displays a broadened stress distribution Å. The localization width increases to *w* = 16.9 Å. While the maximum compressive stress reaches a similar magnitude of −9.3 GPa at the onset of grading, the maximum tensile stress is reduced to 1.6 GPa compared to 2.8 GPa in the sharp interface.

The integrated stress magnitude, representing the total area under the absolute stress curve, was computed as:^19,55,74^4
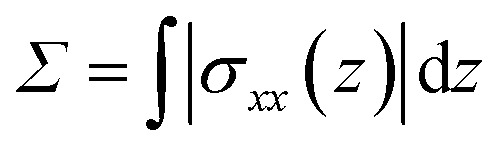


The graded interface yields an integrated stress of *Σ* = 85.62 GPa Å, whereas the sharp interface yields *Σ* = 26.76 GPa Å. This demonstrates that grading redistributes strain over a larger volume rather than intensifying local stress.

### 3.3 Architectural definition and compositional fidelity

**Fig. 4:**
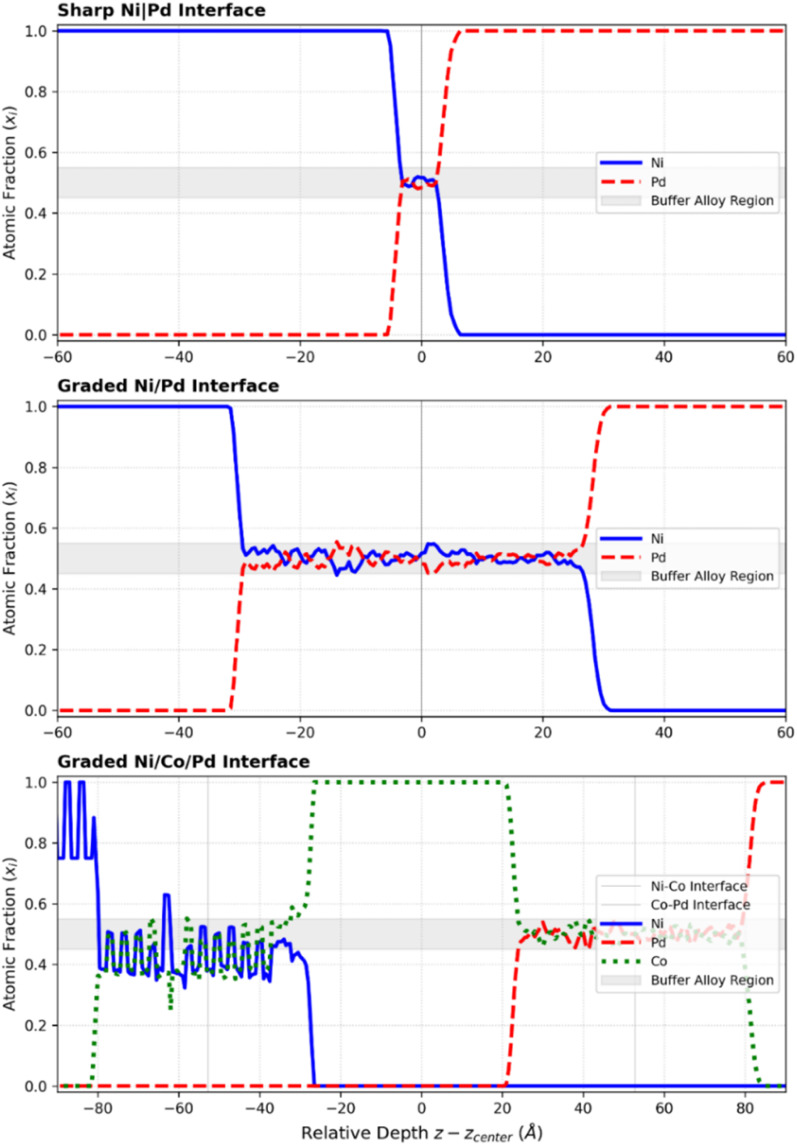
Atomic fraction profiles (*x*_*i*_) *versus* relative depth. (Top) sharp Ni|Pd interface showing abrupt transition. (Middle) graded Ni–Pd interface showing a stable ∼60 Å alloy buffer region at 50% composition. (Bottom) graded Ni–Co–Pd interface showing the separation of Ni and Pd by a central pure Co buffer layer.

To verify that this redistribution arises directly from the intended compositional architecture, the atomic species distribution across the interface is shown in Fig. 4. The profile reveals that the “graded” interface is composed of discrete compositional steps, consistent with the layer-by-layer deposition strategy.

The sharp Ni|Pd interface (top panel) exhibits an abrupt transition width of less than 10 Å, with negligible interdiffusion. The graded Ni–Pd interface (middle panel) reveals a distinct compositional plateau where the atomic fraction of both Ni and Pd remains approximately 0.5 (*x*_Ni_ ≈ *x*_Pd_ ≈ 0.5). This “buffer alloy region” extends over a depth of ∼60 Å (from −30 Å to +30 Å), reflecting the layer-by-layer deposition strategy. The graded Ni–Co–Pd interface (bottom panel) demonstrates the successful integration of a pure Co buffer layer. The profile shows two distinct mixing regions: a Ni–Co interface (left) and a Co–Pd interface (right), separated by a region of pure Co (*x*_Co_ = 1.0) spanning approximately 50 Å. This indicates that the ternary architecture maintains discrete chemical separation between the lattice-mismatched Ni and Pd phases.

### 3.4 Correlation between stress localization and local structural disorder

To examine the structural signature associated with stress redistribution, local lattice distortion was quantified using Voronoi atomic volume analysis. The depth-resolved disorder profile, Δ*σ*_*V*_(*z*), was computed using eqn (2) and represents the local standard deviation of atomic Voronoi volumes within each depth bin.

**Fig. 5:**
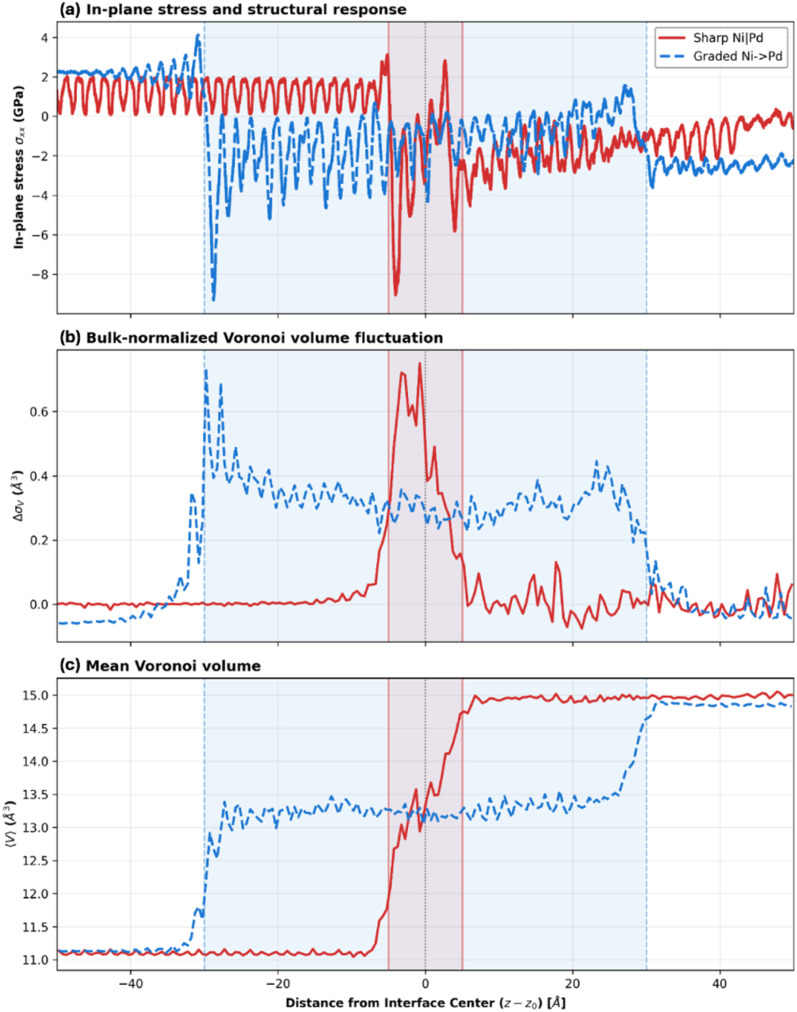
Spatial correlation between mechanical and structural properties for sharp (red solid) and graded (blue dashed) interfaces. (a) In-plane stress *σ*_*xx*_(*z*). (b) Local lattice disorder Δ*σ*_*V*_(*z*) (eqn (2)), showing that the sharp interface has a disorder spike (∼0.75 Å^3^) while the graded interface maintains a lower plateau (∼0.35 Å^3^). (c) Mean Voronoi volume *V*(*z*), illustrating the continuous lattice expansion in the graded architecture.

Fig. 5 presents the spatially resolved correlation between in-plane stress *σ*_*xx*_(*z*) (Fig. 5A), Voronoi volume fluctuation Δ*σ*_*V*_(*z*) (Fig. 5B), and mean atomic volume 〈*V*(*z*)〉 (Fig. 5C). The sharp Ni|Pd interface exhibits a single intense disorder peak reaching Δ*σ*_*V*_(*z*) ≈ 0.75 Å^3^ (Fig. 5B, red solid line), spatially coincident with the sharp stress inversion seen in Fig. 5A. Fig. 5C illustrates an abrupt volumetric jump from ∼11.1 Å^3^ (Ni) to ∼15.0 Å^3^ (Pd) within a narrow region.

On the other hand, the graded Ni–Pd architecture shows a sustained disorder plateau with an average magnitude of Δ*σ*_*V*_(*z*) ≈ 0.35 Å^3^ that extends across the full 60 Å transition region. Fig. 5C demonstrates that the mean atomic volume expands continuously across this region, mitigating the abrupt volumetric mismatch that drives the stress localization in the sharp interface.

**Fig. 6:**
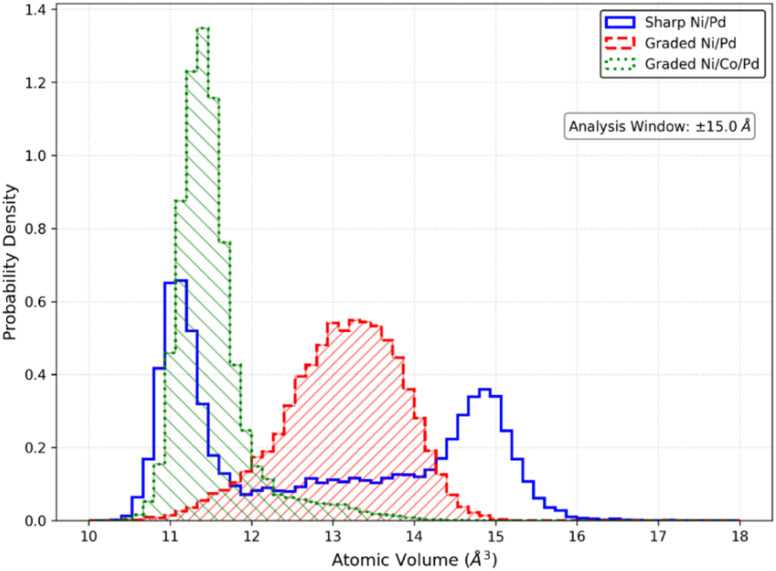
Probability distribution of Voronoi atomic volumes for atoms within the interface region (±15 Å). The sharp interface (blue solid) exhibits a bimodal distribution indicating phase separation. The graded interface (red dashed/hatched) shows a broad, continuous distribution characteristic of effective alloying. The ternary Ni–Co–Pd interface (green dotted) shows a sharp peak corresponding to the ordered Co buffer layer.

To further corroborate this interpretation, the probability distribution of atomic Voronoi volumes within the interface region (±15 Å) is shown in Fig. 6. The sharp interface (blue solid line) displays a distinct bimodal distribution with two separate peaks corresponding to bulk-like Ni and Pd environments. This reflects the coexistence of distinct phases with minimal mixing. Conversely, the graded interface (red dashed line) exhibits a broad, unimodal distribution bridging the gap between the Ni and Pd volumes. This “disorder plateau” suggests that the graded architecture accommodates mismatch through a continuous spectrum of atomic environments rather than discrete defect confinement.

### 3.5 Architectural robustness under increased chemical complexity

**Fig. 7:**
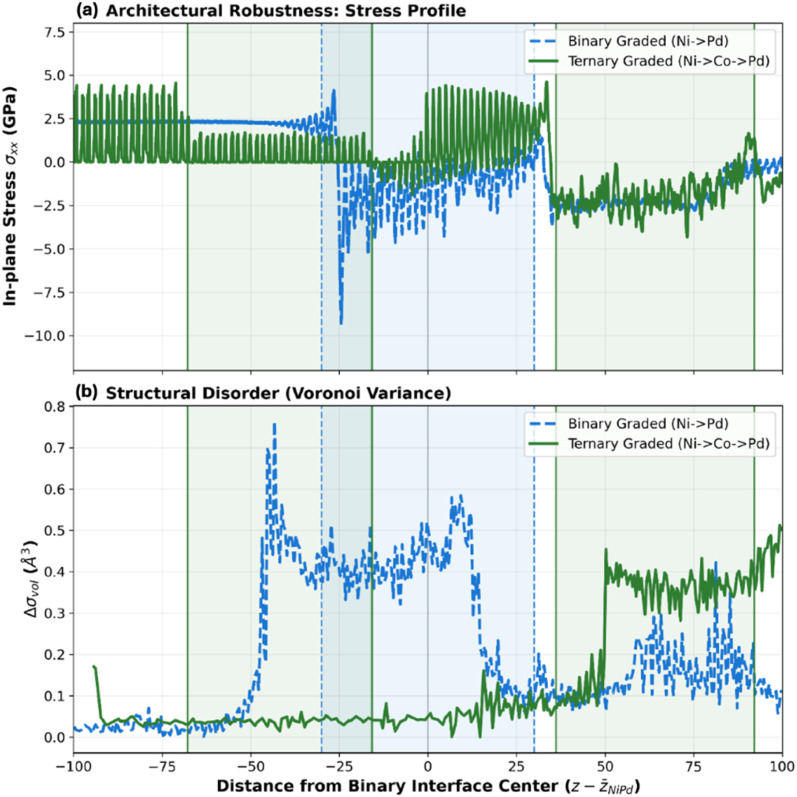
Architectural robustness of the ternary system. (a) In-plane stress profiles show that the ternary design (green) splits the single large stress concentration of the binary system (blue) into two smaller, manageable peaks. (b) Structural disorder profiles reveal that the ternary Co buffer layer creates a region of near-zero lattice distortion (Δ*σ*_*V*_(*z*) < 0.1 Å^3^), unlike the sustained high disorder of the binary alloy.

To assess whether architectural stress redistribution persists under increased chemical complexity, the graded design was extended to a ternary Ni–Co–Pd system. Fig. 7 compares the depth-resolved stress and disorder profiles for the binary graded (blue dashed) and ternary graded (green solid) architectures. Quantitative metrics are summarized in Table 1.

**Table 1:** Quantitative stress and disorder metrics for binary and ternary graded architectures

Metric	Binary graded (Ni–Pd)	Ternary (Ni–Co)	Ternary (Co–Pd)
Window range (*z*′)	−30 to 30 Å	−67.8 to −15.8 Å	36.1 to 92.1 Å
Window center	0 Å	−41.8 Å	64.1 Å
Core peak *σ*_*xx*_ (±10 Å)	4.65 GPa	2.60 GPa	4.34 GPa
Regional max tensile	4.13 GPa	2.60 GPa	1.65 GPa
Regional max compressive	−9.30 GPa	−0.23 GPa	−4.34 GPa
Mean disorder from Δ*σ*_*V*_(*z*) profile	0.35 Å^3^	0.04 Å^3^	0.30 Å^3^
Peak disorder from Δ*σ*_*V*_(*z*) profile	0.76 Å^3^	—	0.51 Å^3^

The ternary architecture effectively decouples the lattice mismatch into two distinct accommodation regions. The Ni–Co transition (left side, *z* ≈ −42 Å) exhibits a low peak stress of 2.60 GPa, consistent with the minimal lattice mismatch (∼0.6%) between Ni and Co. The Co–Pd transition (right side, *z* ≈ 64 Å) accommodates the majority of the lattice expansion, resulting in a peak compressive stress of 4.34 GPa.

Crucially, the maximum compressive stress in the ternary system is approximately 53% lower than that observed in the binary graded Ni–Pd architecture (4.34 GPa *vs.* 9.30 GPa). Fig. 7B shows the structural origin of this improvement. While the binary graded interface maintains a high disorder plateau (Δ*σ*_*V*_(*z*) ≈ 0.35 − 0.50 Å^3^), the ternary system exhibits a “structural quiet zone” corresponding to the pure Co buffer layer where disorder drops to near-bulk levels (<0.05 Å^3^). Significant lattice distortion (Δ*σ*_*V*_(*z*) ≈ 0.30 Å^3^) re-emerges only at the Co–Pd interface, demonstrating that the insertion of a chemically intermediate buffer layer not only spreads the stress field spatially but also suppresses the peak atomic-level distortion.

### 3.6 Defect-mediated relaxation and architectural suppression

The residual coherency stress fields described above coexist with localized defect-like structural signatures. CSP, PTM, and DXA post-processing showed that all three relaxed architectures contain nonzero defect-like content. The systems should therefore not be interpreted as strictly defect-free coherent interfaces. Instead, they are better described as coherency-dominated relaxed interfaces in which residual elastic stress is accompanied by localized HCP-like planar features consistent with stacking-fault-mediated relaxation.^22,26^

The global PTM statistics show a systematic reduction in HCP-like content with increasing architectural complexity. Averaged over the six analysed production-tail frames, the HCP-classified fraction decreased from 14.3% in the sharp Ni|Pd architecture to 10.8% in the graded Ni–Pd architecture and 5.0% in the ternary Ni–Co–Pd architecture. The corresponding FCC-classified fraction increased from 80.0% to 84.6% and 91.5%, respectively. The high-CSP population, defined as atoms with CSP >4.0 Å^2^, also decreased from 18.9% to 14.5% and 8.0%. PTM-derived structural fractions were highly stable across the six analysed frames, with frame-to-frame variation below 0.2 percentage points in all three architectures.

The depth-resolved analysis provides a stronger measure of the engineered interface response. Within the interface or transition region, the mean HCP-classified fraction decreased from 14.7% in the sharp Ni|Pd architecture to 6.2% in the graded Ni–Pd architecture and 1.3% in the ternary Ni–Co–Pd architecture. The corresponding FCC-classified fraction increased from 80.9% to 87.7% and 96.5%. Thus, the ternary architecture produces an approximately eleven-fold reduction in interface-region HCP-like content relative to the sharp Ni|Pd architecture.

DXA detected nonzero line content in all three architectures, further confirming that the systems are not strictly dislocation-free. However, the DXA line length decreased systematically with architectural smoothing. The mean total DXA line length over the six analysed frames decreased from 427.8 nm in the sharp Ni|Pd architecture to 280.5 nm in the graded Ni–Pd architecture and 199.1 nm in the ternary Ni–Co–Pd architecture. DXA line lengths varied modestly across frames, with framewise ranges of 416–438 nm for sharp Ni|Pd, 270–287 nm for graded Ni–Pd, and 189–205 nm for ternary Ni–Co–Pd. The architectural ordering sharp Ni|Pd > graded Ni–Pd > ternary Ni–Co–Pd was preserved in every analysed frame.

In the sharp Ni|Pd system, 98.4% of the DXA line content was located in the interface or transition region. This indicates that the DXA-detected signal originates predominantly from the interfacial region rather than from free-surface artifacts, and it corroborates the PTM-derived conclusion that defect-like content is interface-localized in the sharp architecture. In the graded and ternary systems, the DXA content was lower and more spatially redistributed, consistent with architectural dilution of mismatch-driven relaxation.^24,49^ Final-frame DXA Burgers-vector classification further showed that 1/6〈112〉 Shockley-partial line content dominates the DXA signal in all three architectures, decreasing from approximately 298 nm in the sharp Ni|Pd architecture to 202 nm in the graded Ni–Pd architecture and 134 nm in the ternary Ni–Co–Pd architecture, in proportion to the total DXA line length. This identifies stacking-fault-mediated partial relaxation as the principal mechanism contributing to the DXA-detected line content.^23,44^

In the ternary Ni–Co–Pd architecture, the HCP-like planar features were spatially asymmetric. OVITO visualization showed that the Ni-rich region and Ni–Co transition remained predominantly FCC, while the residual HCP-like features were concentrated mainly toward the Pd-rich side and the Co–Pd transition. This spatial distribution is consistent with the lattice-mismatch hierarchy of the imposed FCC reference structures. The Ni–Co mismatch is small, while the Ni–Pd and Co–Pd mismatches are large.^27,43^ Therefore, the Co buffer does not eliminate all defect-mediated relaxation, but it strongly suppresses HCP-like planar features in the engineered transition region and shifts the residual structural accommodation toward the Pd-rich side.

Together, these results refine the stress-localization interpretation. The architectural benefit of grading and Co buffering is not limited to redistribution of residual coherency stress. The same design strategy also suppresses HCP-like planar defect signatures. Sharp Ni|Pd interfaces localize both stress and defect-like relaxation, graded Ni–Pd interfaces dilute both effects, and ternary Ni–Co–Pd architectures provide the strongest combined reduction in stress localization and stacking-fault-like relaxation.^24,49^

To consolidate the relationship between residual stress localization and structural accommodation, the principal stress metrics were evaluated together with the CSP, PTM, and DXA descriptors across all three architectures. These complementary analyses capture local lattice distortion (CSP), crystallographic environment changes (PTM), and line-defect signatures associated with partial relaxation (DXA). Integrating these quantities allows direct assessment of whether architectural modification alters only the spatial distribution of stress or also changes the underlying accommodation mechanism. Table 2 summarizes the coupled stress–structure response and reveals a consistent architecture-dependent trend: the same design strategies that redistribute coherency stress also reduce HCP-like planar defect signatures, suppress DXA-detectable line content, and increase retention of FCC order within the interface region.

**Table 2:** Coupled stress and defect-mediated relaxation metrics for the three interface architectures. PTM, CSP, and DXA metrics were averaged over six analysed production-tail frames. The results show that compositional grading and ternary Co buffering reduce global HCP-like content, interface-region HCP-like content, high-CSP population, and DXA-detectable line content

Architecture	Peak *σ*_*xx*_ compressive/tensile (GPa)	Mean global HCP (%)	Interface-region HCP (%)	Interface-region FCC (%)	Mean CSP >4 Å^2^ (%)	Mean DXA line length (nm)
Sharp Ni|Pd	−9.1/2.8	14.3	14.7	80.9	18.9	427.8
Graded Ni–Pd	−9.3/1.6	10.8	6.2	87.7	14.5	280.5
Ternary Ni–Co–Pd	−4.34/1.65	5.0	1.3	96.5	8.0	199.1

### 3.7 Summary of architecture-dependent stress fields

Across all architectures, stress localization patterns depend strongly on the spatial distribution of compositional change. Sharp interfaces concentrate coherency strain within narrow regions (±5 Å), whereas graded architectures distribute the same total mismatch over extended volumes (±30 Å).

The newly formalized metrics localization width (*w*), integrated stress magnitude (Σ), and local lattice disorder (Δ*σ*_*V*_(*z*)) collectively demonstrate that architectural width, rather than just chemical composition, governs the spatial distribution of strain energy. While the ternary system demonstrates that chemical buffering can reduce peak stress magnitudes, the fundamental mechanism of redistribution remains geometric. No claims regarding mechanical failure, plastic deformation, or long-term durability are inferred from these elastic stress fields alone; however, the reduction in peak stress and disorder suggests a significantly lower driving force for delamination or defect nucleation.

## 4 Discussion

### 4.1 Critical-thickness context for defect-mediated relaxation

A Matthews–Blakeslee critical-thickness estimate^83,84^ was used to contextualize the observed defect-mediated relaxation. The lattice constants used in constructing the initial Atomsk structures were^85^*a*_Ni_ = 3.52 Å, *a*_Co_ = 3.55 Å, and *a*_Pd_ = 3.89 Å. These values give lattice mismatches of approximately 10.5% for Ni–Pd, 9.6% for Co–Pd, and 0.85% for Ni–Co when referenced to the smaller lattice constant.

For the large Ni–Pd and Co–Pd, the Matthews Blakeslee estimate collapses to the Burgers vector or monolayer scale. These interfaces are therefore well above the thickness range where ideal coherent accommodation would be expected. This is consistent with the critical thickness picture described by Dholabhai and Uberuaga^84^ and Wagner *et al.*,^19^ where coherent growth becomes unstable once mismatch strain can be lowered by misfit dislocation formation. Similar mismatch driven semi coherent accommodation has also been observed atomistically by Sen *et al.*^86^ and AlMotasem *et al.*^87^ using both experimental and numerical techniques. By contrast, the smaller Ni–Co mismatch gives a critical thickness of about 7.2 nm for a representative 60° misfit dislocation geometry, using 
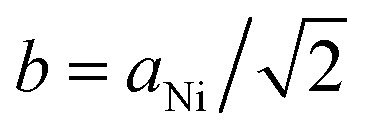
, *ν* = 0.3, and the equation form reported in Table S6.

Since the Ni–Co region is graded rather than abrupt, its effective relaxation threshold should be higher than that of a sharp interface. This agrees with the structural classification obtained using the OVITO framework of Stukowski^80^ and the depth-resolved PTM statistics in Table 2. The ternary engineered interface remains mainly FCC, with only 1.3%, HCP classified atoms, while the remaining HCP like planar features are concentrated toward the Pd rich and Co–Pd side.

The Matthews–Blakeslee model is used here as an order-of-magnitude continuum guide, not as an exact atomistic predictor. It indicates whether elastic coherency is expected to remain stable at the simulated thickness scale. The observed relaxation pathway, namely HCP like planar features consistent with stacking fault mediated relaxation, depends on the local atomic structure and the stacking fault energetics of the EAM potential. This is consistent with Yang *et al.*,^23^ and AlMotasem *et al.*,^87^ who link misfit dislocation dissociation and intrinsic stacking fault formation to local interfacial energetics. Similar atomistic behaviour has been reported by Daghbouj *et al.*,^88^ who showed that local defect evolution and strain accommodation pathways emerge through energetically preferred defect configurations rather than ideal coherent relaxation.

### 4.2 Continuum coherency-energy scale

As an order of magnitude anchor for the simulated stress magnitudes, the continuum coherency strain energy density was estimated as *U*_coh_ ≈ 0.5*M*_eff_*f*^2^, where *f* is the lattice mismatch and *M*_eff_ = *E*_eff_(1 − *ν*_eff_)^−1^ is the effective biaxial modulus. *E*_eff_ was approximated using the harmonic mean of the elemental Young moduli and representative room temperature elastic constants. Using the same construction lattice constants, the estimated coherency energy density is approximately 1.28 GJ m^−3^ for Ni–Pd and 1.08 GJ m^−3^ for Co–Pd, but only 0.011 GJ m^−3^ for Ni–Co. The Pd containing interfaces therefore have coherency energy scales about two orders of magnitude larger than Ni–Co, consistent with the mismatch driven elastic energy discussed by Sen *et al.*^86^

Multiplying these energy densities by the corresponding accommodation widths gives integrated scales of approximately 5.1 GPa Å for the sharp Ni–Pd interface over 4 Å, 67.7 GPa Å for the 52.8 Å graded Ni–Pd ramp, 0.57 GPa Å for the 52.8 Å Ni–Co ramp, and 53.8 GPa Å for the 49.7 Å Co–Pd ramp as summarized in Table S7. These values are continuum scale indices rather than direct predictions of the virial stress integral. As discussed by Parthasarathy *et al.*,^82^ finite temperature atomistic stress calculations include atomic scale relaxation and thermal contributions that are absent from static continuum estimates. The continuum values nevertheless show that Ni–Pd and Co–Pd mismatch strains can support GPa level residual stresses, while Ni–Co is intrinsically much weaker. This explains why the stress and HCP like planar defect signatures shift away from the Ni rich and Ni–Co side and toward the Pd rich and Co–Pd side, consistent with the mismatch-controlled defect accommodation described by Yang *et al.*,^27^ Sen *et al.*,^86^ and AlMotasem *et al.*^87^

A complementary fully coherent stress scale follows from *σ*_coh_ ≈ *Mf*. For Ni–Pd, representative biaxial moduli give stresses of about 20 to 30 GPa, which are higher than the relaxed residual stress peaks. This difference indicates substantial partial relaxation through the PTM and DXA identified HCP like stacking fault mechanisms. Like the semi coherent FCC interfaces described by Shao *et al.*^18^ and Xiang *et al.*,^26^ and the stacking fault mediated relaxation reported by AlMotasem *et al.*,^87^ the relaxed interface should be viewed as a stress reduced state produced by misfit networks and stacking fault mediated accommodation. It should not be interpreted as a direct percentage of strain energy release.

### 4.3 Geometric dilution of the coherency strain field

A central finding of this work is that interfacial stress is controlled not only by lattice mismatch, but also by the width over which that mismatch is accommodated. In Fig. 3, the sharp Ni|Pd interface confines the full ∼10.5% mismatch within 4.4 Å, giving a localized compressive peak of −9.1 GPa. This is the expected response of a semi-coherent boundary, where abrupt misfit networks act as stress concentrators and favour local defect activity, as described by Chen *et al.*^24^ and Chauniyal and Janisch.^44^ Similar mismatch driven stress localization has also been reported by Sen *et al.*^86^ and AlMotasem *et al.*^87^ for sharply defined metallic multilayers. The screening in Section 3.6 shows that this interface also has the highest stacking fault like defect content among the three systems. This links the residual stress maximum to local partial relaxation, consistent with the HCP marked intrinsic stacking fault regions reported by Xiang *et al.*^26^ and AlMotasem *et al.*^87^

The graded architecture changes this condition by spreading the compositional transition across ∼60 Å (Fig. 4). Instead of concentrating misfit at one plane, the graded interface distributes it across several atomic layers. The integrated stress sum therefore increases to 85.62 GPa Å because a larger region contributes to strain accommodation, while the peak tensile stress decreases by ∼43% from 2.8 to 1.6 GPa. Grading therefore does not remove the mismatch. It geometrically dilutes the local stress field by converting a sharp stress raiser into a broader accommodation zone. This interpretation agrees with Chen *et al.*,^24^ who showed that three-dimensional chemical gradients diffuse interfacial stress concentrations, and with Zhu *et al.*,^49^ who linked compositional undulation to stress and strain delocalization in FCC alloys. The reduction in interface-region HCP fraction from 14.7% to 6.2% (Table 2) further confirms that the lower peak stress is accompanied by weaker localized stacking-fault-like accommodation.

### 4.4 Structural disorder: the atomistic signature of grading

The mechanism of this accommodation is reflected in the stress and local structural disorder correlation shown in Fig. 5. For the sharp interface, the stress inversion in Fig. 5A coincides with a disorder spike of Δ*σ*_*V*_(*z*) ≈ 0.75 Å^3^ in Fig. 5B. Atoms at the phase boundary therefore occupy highly distorted local environments as they bridge the Ni and Pd lattice gap. This behaviour follows the local lattice distortion reported by Lu *et al.*^89^ near misfit dislocation cores, the mismatch related elastic energy described by Sen *et al.*,^86^ and the link between atomistic stress and Voronoi volume disruption described by Li and Chew.^71^ The bimodal Voronoi volume distribution in Fig. 6 supports this interpretation, since the two peaks correspond to strained Ni like and Pd like environments separated by an atomically narrow transition.

The graded architecture instead develops a lower disorder plateau of Δ*σ*_*V*_(*z*) ≈ 0.35 Å^3^ across the transition region. Together with the unimodal Voronoi volume distribution in Fig. 6, this plateau indicates distributed volumetric strain accommodation rather than confinement of mismatch to one plane. Like the gradual Voronoi index variation used by Alishahi and Deng^70^ to identify smooth structural transitions, the graded system accommodates mismatch through smaller local volume adjustments across several atomic layers. This mechanism agrees with Chen *et al.*,^24^ who showed that chemical gradients spread lattice mismatch over several nanometers, and with Zhu *et al.*,^49^ who linked compositional undulation to strain delocalization. The lower HCP classified and high CSP populations in Section 3.6 therefore confirm that grading reduces localized stacking fault like accommodation.

### 4.5 Decoupling strain *via* chemical buffering in ternary systems

Binary grading spreads the mismatch geometrically, while the ternary Ni–Co–Pd architecture further separates the strain field chemically. As shown in Fig. 7A, inserting Co divides the single Ni–Pd accommodation region into two spatially separated transitions, Ni–Co and Co–Pd. This partitioning lowers the maximum compressive stress by 53% relative to the binary graded architecture, from 9.30 to 4.34 GPa (Table 1).

The structural origin of this improvement is the quiet zone in Fig. 7B, where Δ*σ*_*V*_(*z*) approaches near bulk values. The Co layer therefore acts as a mechanically intermediate buffer that weakens direct coupling between the Ni rich and Pd rich expansion regimes. This role is similar to the Fe interlayer studied by Pang *et al.*,^90^ where an intermediate layer partitions a mismatched interface and attenuates stress concentration associated with defect transmission. It also follows the strain compensation mechanism reported by Daghbouj *et al.*^86,91,92^ experimental and numerical studies, where oppositely distorted regions reduce the net residual strain. The continuum estimates in Section 4.2 support the same interpretation, since the Ni–Co coherency energy density is nearly two orders of magnitude lower than the Ni–Pd and Co–Pd values. The defect screening in Section 3.6 follows this trend, with the ternary system showing the lowest interface region HCP fraction and the lowest mean DXA line length.

The Co buffer should therefore be interpreted as a strain partitioning layer rather than a simple chemical insertion. It does not remove all mismatch driven relaxation, but it suppresses HCP like planar features in the engineered transition region and shifts the remaining accommodation toward the Pd rich and Co–Pd side. This agrees with AlMotasem *et al.*,^87^ where interfaces hinder dislocation extension and suppress loop formation, and with Zhu *et al.*^49^ who linked compositional modulation to stress and strain delocalization. The ternary design therefore shows that intermediate lattice parameter buffers can reduce both peak residual stress and partial relaxation signatures for mismatches near 10%.

### 4.6 Implications for hydrogen environment embrittlement

The stress landscapes mapped here are relevant to hydrogen-coupled degradation, although hydrogen was not included explicitly in the present simulations. In FCC metals, Rao *et al.*^93^ and Psarras *et al.*^94^ show that tensile stress fields and local lattice dilation can favour hydrogen trapping. The sharp Ni|Pd interface therefore represents a plausible accumulation site once hydrogen transport is introduced, since it combines a tensile peak of 2.8 GPa with local Voronoi volumes above 15 Å^3^ (Fig. 3 and 5C). Through experimental and numerical methods, similar stress assisted gas accumulation has also been reported by Liu *et al.*,^95^ although in a non-hydrogen irradiation context. Such locally dilated tensile regions may lower resistance to decohesion or promote plasticity assisted failure, consistent with the hydrogen degradation pathways discussed by Psarras *et al.*^94^ and Rao *et al.*.^93^

The graded and ternary architectures reduce this risk by weakening the local mechanical driving force. Binary grading lowers the tensile peak to 1.6 GPa and removes the abrupt volume jump seen in Fig. 5C. The ternary architecture adds a low disorder Co rich region in Fig. 7B, which may separate hydrogen-sensitive zones from the strongest mismatch driven distortion. This interpretation is consistent with the strain compensation mechanisms described by Sen *et al.*^86^ and Daghbouj *et al.*,^91^ where opposite local distortions reduce the net strain field. The stacking fault like features identified in Section 3.6 may also affect hydrogen transport, since Alí *et al.*^30^ showed that planar defects and boundary regions in Pd can alter hydrogen accumulation and mobility. More broadly, Daghbouj *et al.*^96^ showed that engineered boundaries can act as gas trapping and defect sink regions, supporting the need to include explicit gas transport in future simulations. These implications require explicit hydrogen simulations. The present results define the mechanical and structural baseline for those future calculations.

### 4.7 Scope and limitations

This study isolates intrinsic lattice level stress redistribution and defect mediated relaxation under idealized single crystal and hydrogen free conditions. The equilibration data in Fig. 2 confirm a stable 300 K baseline for comparing the three architectures. Grain boundaries, surface roughness, preexisting dislocation networks, hydrogen diffusion, and chemical reactions were not included. Since Alí *et al.*,^30^ Hachet *et al.*^97^ show that microstructural features can act as hydrogen sensitive trapping and stress concentration sites and Daghbouj *et al.*^96^ showed related boundary mediated gas trapping in an irradiation context. The stress fields reported here should be viewed as a post fabrication mechanical baseline rather than a direct prediction of membrane service life.

The stress reductions in Tables 1 and 2 indicate a lower local driving force for defect formation and interfacial degradation, but they do not determine long-term durability by themselves. This interpretation is consistent with Sen *et al.*,^86^ who linked lower elastic strain to reduced defect growth. Hydrogen transport, high temperature diffusion, intermetallic formation, external loading, and temperature dependent defect energetics remain outside the present model. These effects should be included in future hydrogen-coupled and thermomechanical simulations, particularly because Liang *et al.*^5^ and Park *et al.*^8^ show that diffusion, phase evolution, and intermetallic growth can control degradation in Pd based composite membranes. Broadly, Daghbouj *et al.*^98^ showed that defect landscape engineering can limit gas damage in ceramics, supporting future coupled hydrogen transport and defect evolution simulations.

All molecular dynamics simulations were performed at 300 K, so differential thermal expansion was not simulated in the reported stress profiles. A first order estimate based on linear thermal expansion coefficients gives differential thermal strains per 100 K above 300 K of 0.016% for Ni–Pd, 0.012% for Co–Pd, and 0.004% for Ni–Co (Table S8). These values are small compared with the lattice mismatches of 10.5%, 9.6%, and 0.85%. The hierarchy Ni–Pd ≈ Co–Pd ≫ Ni–Co is therefore preserved. Although Peters *et al.*^6^ and Abedini *et al.*^99^ show that thermal expansion mismatch can generate residual stress during membrane thermal cycling, the present estimate indicates that intrinsic lattice mismatch remains the dominant source of local atomic scale distortion in this 300 K baseline. The predicted stress and disorder fields provide testable targets for future experiments, including nanobeam electron diffraction, 4D-STEM strain mapping, related TEM diffraction methods, high-resolution X-ray diffraction, and reciprocal-space mapping of deposited Ni–Pd or related multilayer systems.

## 5 Conclusion

This study demonstrates that interface architecture provides a powerful mechanism for controlling residual coherency stress and defect-mediated relaxation in lattice-mismatched Ni–Pd membrane interfaces. Atomistic simulations show that a sharp Ni|Pd interface confines the ∼10.5% lattice mismatch within an atomically narrow region, generating highly localized stress peaks of approximately −9.1 GPa compressive and 2.8 GPa tensile. CSP, PTM, and DXA post-processing further show that this sharp architecture also contains the highest HCP-like planar defect content, with a mean global HCP-classified fraction of 14.3% and a mean DXA line length of 427.8 nm. In contrast, a compositionally graded Ni–Pd interface distributes the mismatch across a wider transition zone, reducing the peak tensile stress by approximately 43% and lowering the mean global HCP-classified fraction to 10.8%. Introducing a Co buffer layer in a ternary Ni–Co–Pd architecture further modifies the stress landscape by separating the mismatch into two accommodation regions and reducing the peak compressive stress by approximately 53% relative to the binary graded system. The ternary architecture provides the strongest suppression of defect-like structural signatures, reducing the mean global HCP-classified fraction to 5.0% and the interface-region HCP-classified fraction to 1.3%. These results show that geometric grading and intermediate buffer layers suppress both residual coherency-stress localization and HCP-like stacking-fault-mediated relaxation. Nanoscale grading and chemically intermediate buffer layers therefore provide effective architectural strategies for improving structural stability in lattice-mismatched multilayer membranes.

## Conflicts of interest

The authors declare no conflicts of interest.

## Supplementary Material

NA-OLF-D6NA00262E-s001

## Data Availability

The data supporting this article are included in the main article and in the supplementary information (SI). Supplementary information: stress-reconstruction and global pressure consistency validation, temporal convergence analysis, spatial-binning robustness checks, defect-mediated relaxation screening using CSP, PTM and DXA, Matthews–Blakeslee critical-thickness estimates, continuum coherency strain-energy estimates and first-order thermal-expansion sensitivity estimates. Additional simulation input files, processed stress-profile data and post-processing data are available from the corresponding author upon reasonable request. See DOI: https://doi.org/10.1039/d6na00262e.
