# Supplementary material for: Nanoscale control of coherency stress in Ni–Pd interfaces through grading and ternary buffer layers
Source: Nanoscale Adv. 2026 Jun 26;8(15):4330–46. doi: 10.1039/d6na00262e (PMC13339988; doi:10.1039/d6na00262e)
Supplement: NA-008-D6NA00262E-s001 [file NA-008-D6NA00262E-s001.pdf]

Supplementary Information (SI) for Nanoscale Advances.
This journal is © The Royal Society of Chemistry 2026

Supporting Material

Nanoscale Control of Coherency Stress in Ni–Pd Interfaces through Grading and Ternary Buffer Layers

Emanuel Peters-Teke Tebo [11], Sina Karimzadeh [12], Tien-Chien Jen [13*]

[1] *Department of Mechanical Engineering Science, University of Johannesburg, Gauteng, 2006, South Africa*

Contents

S1. Stress reconstruction and global consistency validation 2

S2. Temporal convergence of stress profiles 3

S2.1 Spatial robustness with respect to binning resolution 4

S3. Defect-mediated relaxation screening 4

Figures: S1, S2, S3. Tables: S1–S8. Equations: S1–S6.

[1] Email: 221191307@student.uj.ac.za

[2] Email: skarimzadeh@uj.ac.za

[3]* Corresponding Author, Email: tjen@uj.ac.za

S1. Stress reconstruction and global consistency validation

To confirm the mechanical fidelity of the depth-resolved stress profiles used in the main text, spatially resolved virial stress profiles were integrated to reconstruct the global pressure tensor components. This reconstructed pressure was compared against the thermodynamic pressure reported by the molecular dynamics engine to ensure macroscopic momentum balance.

The reconstructed pressure component was computed as:

$$p_{\alpha\alpha}^{rec}(t) = -\frac{\sum_{bins} S_{(\alpha\alpha,sum)}(z,t)}{V(t)} \qquad (S1)$$

where $S_{(\alpha\alpha,sum)}(z,t)$ is the per-bin virial stress sum (units: bar·Å^3) obtained from the spatial binning, and $V(t) = L_x(t)L_y(t)L_z(t)$ is the instantaneous simulation cell volume.

The instantaneous reconstruction error was defined as:

$$e_{\alpha\alpha}(t) = p_{\alpha\alpha}^{rec}(t) - p_{\alpha\alpha}(t) \qquad (S2)$$

where $p_{\alpha\alpha}(t)$ is the corresponding thermodynamic pressure component output by the simulation engine.

Regression analysis: Because the transverse components (P_{xx} and P_{yy}) are barostat-controlled and fluctuate near zero, correlation-based metrics can appear artificially weak. Therefore, two complementary validation criteria were employed:

i. **Normal component (P_{zz}):** Linear regression analysis (slope, coefficient of determination R^2, and Pearson correlation coefficient r).

ii. **Transverse components (P_{xx}, P_{yy}):** Block-averaged mean error criterion. We verify that the mean error is statistically indistinguishable from zero using the standard error (SE):

$$|\bar{e}| \leq 2 \cdot SE(\bar{e}) \qquad (S3)$$

where $\bar{e}$ is the mean error averaged over blocks of 25 samples (following the method of Flyvbjerg & Petersen, 1989). This criterion ensures that the reconstruction error is within the bounds of thermal noise.

Table S1 summarizes the results. For the mechanically relevant normal component P_{zz}, regression analysis yielded near-identity agreement across all architectures, with slopes ranging from 0.976 to 0.998 and $R^2 \geq 0.980$. For the transverse components, block-averaged reconstruction errors remained statistically negligible, with mean bias values $|\bar{e}| \leq 1.16$ bar relative to standard errors of approximately $0.60 - 0.96$ bar.

Table S1. Global pressure tensor consistency between thermodynamic output and reconstructed pressures. Block mean statistics were computed using 25-sample blocks over 400 overlapping samples. **Note:** Regression metrics (R^2, Slope) are only informative for the normal component (P_{zz}); transverse components are dominated by thermal noise around the barostat setpoint.

System	Component	Regression slope	R^2	Pearson r	Block mean error (bar)	Block stderr (bar)
Graded	P_{xx}	0.131848	0.051623	0.227208	-0.05721	0.962467
	P_{yy}	0.193291	0.111808	0.334377	0.813011	0.767223
	P_{zz}	0.976441	0.979886	0.989892	0.315259	0.740483
Ternary	P_{xx}	0.117603	0.042338	0.205763	0.687320	0.706876
	P_{yy}	0.147626	0.060176	0.245309	-0.032241	0.624465
	P_{zz}	0.994717	0.995832	0.997914	-0.300838	0.593227
Sharp	P_{xx}	0.130589	0.041813	0.204483	1.061340	0.597626
	P_{yy}	0.129238	0.043979	0.209713	1.157940	0.628120
	P_{zz}	0.997661	0.998066	0.999033	1.418330	0.555079

S2. Temporal convergence of stress profiles

To ensure thermodynamic stability of the depth-resolved stress profiles, convergence was assessed by varying the time averaging window ($50, 100,$ and 200 ps). The instantaneous interface position $z_0(t)$ was dynamically tracked using a composition gradient criterion:

$$z_0(t) = \underset{z}{arg,max}\left|\frac{dx_{Ni}(z,t)}{dz}\right| \qquad (S4)$$

where the term $\underset{z}{arg,max}$ identifies the depth z where the composition gradient is steepest (the center of the interface). The local Ni atomic fraction x_{Ni} is defined as:

$$x_{Ni} = \frac{N_{Ni}}{N_{Ni} + N_{Co} + N_{Pd}} \qquad (S5)$$

Table S2 summarizes the convergence metrics relative to the 200 ps reference window. Graded interfaces converged rapidly, with peak stress deviations below 2.2% at 100 ps. In contrast, the sharp Ni|Pd interface exhibited slower relaxation due to the abrupt compositional discontinuity, with peak deviations exceeding 3% at 100 ps. Consequently, a conservative 200 ps averaging window was adopted for all reported analyses.

Table S2. Temporal convergence metrics relative to the 200 ps reference window

System	Δpeak (%) at 50 ps	Δpeak (%) at 100 ps	RMS diff (GPa) at 100 ps	Physical interpretation
Graded	0.08	0.32	0.011	Smooth compositional gradient promotes rapid stress decorrelation
Ternary	0.22	2.19	0.018	Multicomponent broadening increases temporal fluctuations
Sharp	5.39	3.88	0.054	Abrupt interface induces slow stress relaxation

S2.1 Spatial robustness with respect to binning resolution

Sensitivity to spatial discretization was evaluated by rebinning stress profiles from a native resolution of $\Delta z \approx 0.5$ Å to coarser resolutions up to 2.0 Å using virial-conserving aggregation. While coarser binning attenuates peak stress magnitudes due to spatial smoothing, the stress centroid position remained invariant:

$$z_{centroid} = \frac{\sum|\sigma_{xx}(z)|\, z}{\sum|\sigma_{xx}(z)|} \qquad (S6)$$

where $\sigma_{xx}(z)$ is the depth-resolved in-plane stress component and z denotes the bin center coordinate.

Maximum centroid shifts were below 0.4 bins (< 0.8 Å) across all systems (Table S3), confirming that observed stress localization features are physical consequences of interface architecture rather than numerical artifacts of binning resolution.

Table S3. Spatial robustness of near-interface stress localization for graded Ni-Co-Pd under virial-conserving rebinning

Bin Width Δz (Å)	Peak |σxx| (GPa)	z_centroid (Å)	Absolute Shift |Δz_centroid| (Å)	Normalized Shift (bins)
0.5 (Ref)	1.31	62.72	—	—
1.0	0.65	62.74	0.02	0.02
2.0	0.63	61.92	0.8	0.4

S3. Defect-mediated relaxation screening

Representative production-tail snapshots were post-processed in OVITO to quantify local defect-mediated relaxation. Three complementary descriptors were used. CSP was computed using 12 nearest neighbours to screen local lattice distortion. PTM was used to classify atomic environments as FCC, HCP, BCC, ICO, or Other. DXA was performed using an FCC reference lattice to identify line-defect content and Burgers-vector categories.

PTM was used as the primary metric for stacking-fault-like planar relaxation because HCP-classified atomic sheets in an FCC matrix indicate local interruptions of the close-packed stacking sequence. DXA was treated as a corroborating diagnostic because the detected line content includes multiple Burgers-vector categories, with $1/6\langle 112\rangle$ Shockley-type line content forming the dominant contribution in the representative final-frame breakdown.

To distinguish interfacial defect signatures from free-surface effects, depth-resolved binning was performed along the interface-normal z-direction. Atoms near the shrink-wrapped free surfaces were analysed separately from interface or transition regions and bulk-like interior regions. The global statistics are summarized in Table S4, while the depth-resolved regional statistics are summarized in Table S5. The Matthews–Blakeslee estimates used to contextualize the observed relaxation are summarized in Table S6. Representative PTM maps are shown in Figure S1, CSP histograms are shown in Figure S2, and DXA Burgers-vector breakdowns are shown in Figure S3.

Table S4. Global CSP, PTM, and DXA defect-screening statistics for the sharp Ni|Pd, graded Ni–Pd, and ternary Ni–Co–Pd architectures. CSP was computed using 12 nearest neighbours, PTM was used to quantify FCC, HCP, and Other structural fractions, and DXA was performed using an FCC reference lattice. The results show nonzero defect-like content in all three relaxed architectures, with systematic decreases in HCP fraction, high-CSP population, and DXA line length from sharp Ni|Pd to graded Ni–Pd and ternary Ni–Co–Pd

**Architecture**	**Frames analysed**	**Mean FCC (%)**	**Mean HCP (%)**	**HCP range (%)**	**Mean Other (%)**	**Mean CSP > 4 Å² (%)**	**Mean DXA line length (nm)**	**DXA range (nm)**
Sharp	6	80.0	14.3	14.22–14.34	5.7	18.9	427.8	416.2–438.1
Graded	6	84.6	10.8	10.76–10.90	4.5	14.5	280.5	269.7–287.1
Ternary	6	91.5	5.0	4.96–5.14	3.4	8.0	199.1	189.2–205.0

Table S5. Depth-resolved CSP, PTM, and DXA statistics for the interface or transition region. The sharp Ni|Pd architecture concentrates HCP-like and DXA-detectable defect signatures in the interface or transition region, with 98.4% of the DXA line content located in that region. The graded Ni–Pd architecture reduces and redistributes this defect-like content. The ternary Ni–Co–Pd architecture shows the lowest HCP-like fraction in the interface or transition region, with the Ni-rich and Ni–Co regions remaining predominantly FCC and the residual HCP-like features concentrated mainly toward the Pd-rich and Co–Pd side

**Architecture**	**Interface-region FCC (%)**	**Interface-region HCP (%)**	**Interface-region DXA line length (nm)**	**DXA line content in interface region (%)**	**Main depth-resolved observation**
Sharp	80.9	14.7	421.0	98.4	Defect-like content is primarily interface-localized rather than free-surface dominated.
Graded	87.7	6.2	141.3	50.4	Defect-like content is reduced and more spatially redistributed.
Ternary	96.5	1.3	108.3	54.4	Engineered transition region is predominantly FCC, with residual HCP-like content shifted toward the Pd-rich and Co–Pd side.

Table S6. Matthews–Blakeslee critical-thickness estimates for the FCC Ni–Pd, Ni–Co, and Co–Pd interfaces constructed in the simulations. The lattice constants used in the initial Atomsk construction were $a_{Ni} = 3.52$ Å, $a_{Co} = 3.55$ Å, and $a_{Pd} = 3.89$ Å. The Ni–Pd and Co–Pd mismatches are approximately 10.5% and 9.6%, respectively, which place the estimated critical thickness at the Burgers-vector or monolayer scale. The Ni–Co mismatch is approximately 0.85%, giving $h_c \approx 7.2$ nm for a representative 60° misfit-dislocation geometry. The Ni–Co estimate was computed using $b = a_{Ni}/\sqrt{2} = 2.49$ Å, $\nu = 0.3$, $\alpha = 60^{\circ}$, and $\cos\lambda = 0.5$. The implicit equation $h_c = [b(1 - \nu\cos^2\alpha)/(8\pi f(1 + \nu)\cos\lambda)][\ln(h_c/b) + 1]$ was solved iteratively. These values are used as order-of-magnitude guidance because the continuum Matthews–Blakeslee model becomes approximate when the calculated critical thickness approaches an atomic-layer scale

Interface	Initial lattice constants used (Å)	Mismatch, f(%)	Burgers vector used	Estimated h_c	Interpretation
Ni–Pd	$a_{Ni} = 3.52$, $a_{Pd} = 3.89$	10.5	$b = a_{Ni}/\sqrt{2}$ =	Burgers-vector / monolayer scale	Large mismatch places the interface far above the critical-thickness scale for ideal coherent accommodation.
Ni–Co	$a_{Ni} = 3.52$, $a_{Co} = 3.55$	0.85	$b = a_{Ni}/\sqrt{2}$ =	≈7.2 nm	Small mismatch supports near-coherent accommodation, especially because the Ni–Co region is compositionally graded rather than abrupt.
Co–Pd	$a_{Co} = 3.55$, $a_{Pd} = 3.89$	9.6	$b = a_{Co}/\sqrt{2}$ =	Burgers-vector / monolayer scale	Large mismatch explains why residual HCP-like features are concentrated toward the Pd-rich and Co–Pd side.

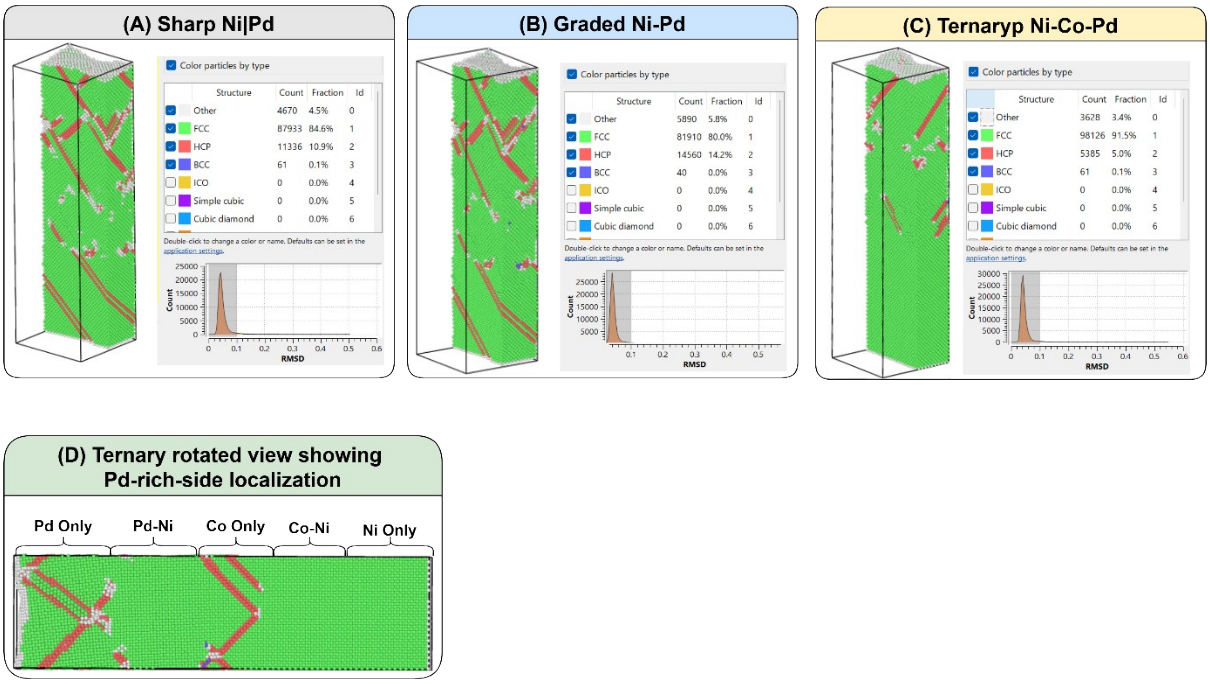

Figure S1. PTM visualization of HCP-like planar defect signatures in the relaxed interface architectures. FCC atoms are shown in green, HCP-classified atoms in red, and PTM-classified Other atoms in white. The sharp Ni|Pd architecture shows the most extensive HCP-like planar content, the

graded Ni–Pd architecture shows reduced HCP-like content, and the ternary Ni–Co–Pd architecture shows the lowest HCP-like content in the engineered interface or transition region. In the ternary architecture, the residual HCP-like features are spatially asymmetric and concentrated mainly toward the Pd-rich and Co–Pd side.

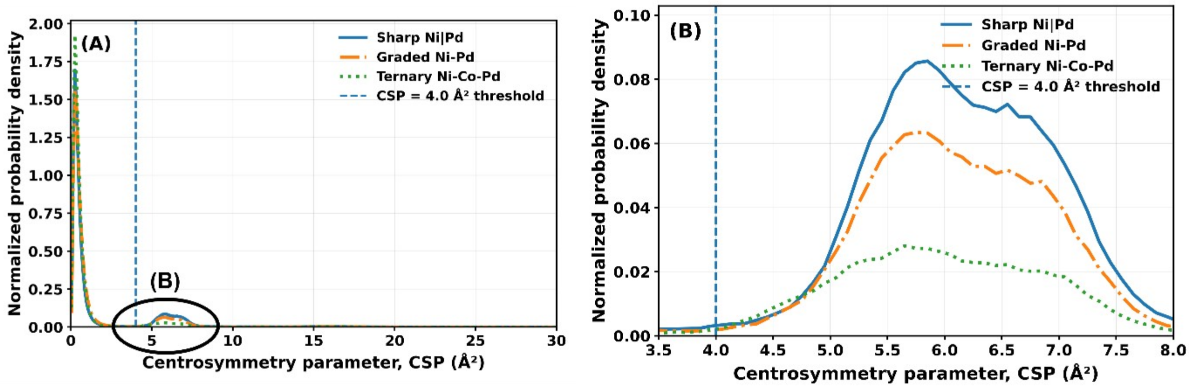

Figure S2. CSP distributions for the sharp Ni|Pd, graded Ni–Pd, and ternary Ni–Co–Pd architectures. CSP was computed using 12 nearest neighbours. The high-CSP population decreases systematically from the sharp Ni|Pd architecture to the graded Ni–Pd architecture and the ternary Ni–Co–Pd architecture, confirming that architectural smoothing reduces local lattice distortion

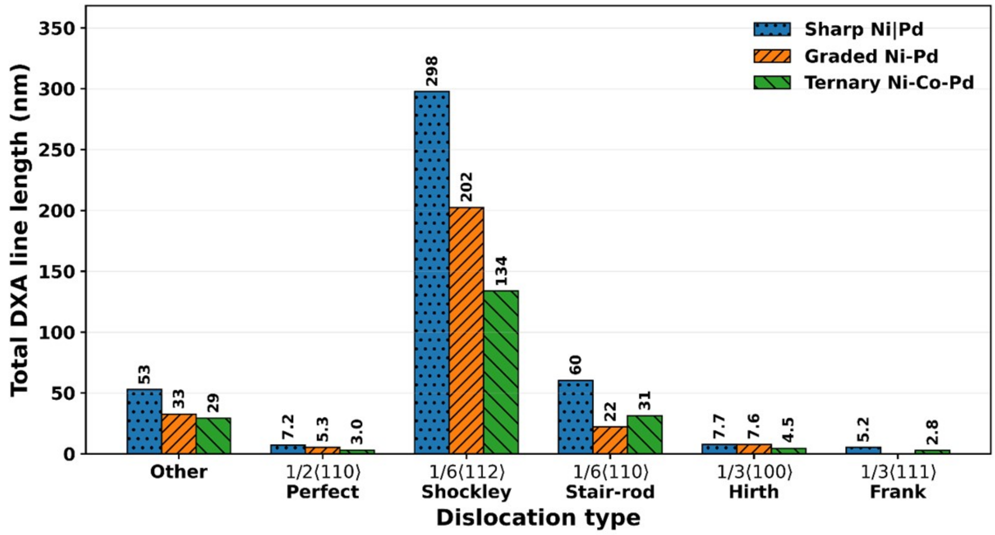

Figure S3. Representative final-frame DXA Burgers-vector breakdown for the relaxed interface architectures. DXA was performed using an FCC reference lattice, and total line length is reported in nm. DXA detected nonzero line content in all three systems, with the largest contribution assigned to $1/6\langle 112\rangle$Shockley-type line content. The total DXA line content decreases systematically from sharp Ni|Pd to graded Ni–Pd and ternary Ni–Co–Pd, confirming that architectural grading and Co buffering suppress line-defect signatures.

Table S7. Continuum coherency strain-energy estimates for the FCC Ni–Pd, Ni–Co, and Co–Pd interfaces. The energy density was estimated using $U_{coh} \approx \frac{1}{2} M_{eff} f^2$, where $M_{eff} = E_{eff}/(1 - \nu_{eff})$. The harmonic mean of the elemental Young's moduli was used for E_{eff}. The integrated scale $U_{coh} w$ is reported in GPa Å using the corresponding accommodation width w. These values provide an order-of-

magnitude continuum comparison and should not be interpreted as direct predictions of the atomistic virial-stress integral.

Region		Mismatch f(%)	E_{eff}(GPa)	M_{eff}(GPa)	U_{coh}(GJ m⁻³)	Width w(Å)	$U_{coh}w$(GPa Å)
Sharp interface	Ni–Pd	10.51	150.8	231.9	1.28	4.0	5.1
Graded ramp	Ni–Pd	10.51	150.8	231.9	1.28	52.8	67.7
Ternary ramp	Ni–Co	0.85	204.4	296.2	0.011	52.8	0.57
Ternary ramp	Co–Pd	9.58	153.3	235.8	1.08	49.7	53.8

Table S8. First-order differential thermal-expansion sensitivity estimate relative to the 300 K simulation baseline. All MD simulations in this work were performed at 300 K, so these values are not simulation results. The differential thermal strain is reported per 100 K above 300 K using $\epsilon_{th} = |\alpha_A - \alpha_B|\Delta T$, with $\alpha_{Ni} = 13.4 \times 10^{-6}\,\mathrm{K}^{-1}$, $\alpha_{Co} = 13.0 \times 10^{-6}\,\mathrm{K}^{-1}$, and $\alpha_{Pd} = 11.8 \times 10^{-6}\,\mathrm{K}^{-1}$. The small thermal increments do not alter the lattice-mismatch hierarchy.

Region	$\lvert\Delta\alpha\rvert$ (10^{-6} K^{-1})	εth per 100 K (%)	Lattice mismatch (%)	Thermal strain per 100 K / mismatch (%)	Interpretation
Ni–Pd	1.6	0.016	10.51	0.15	Thermal expansion is minor relative to the lattice mismatch.
Ni–Co	0.4	0.004	0.85	0.47	Thermal expansion remains small relative to the lattice mismatch.
Co–Pd	1.2	0.012	9.58	0.13	Thermal expansion is minor relative to the lattice mismatch.
